# Complete Low-Cost Implementation of a Teleoperated Control System for a Humanoid Robot

**DOI:** 10.3390/sl30201385

**Published:** 2013-01-24

**Authors:** Andrés Cela, J. Javier Yebes, Roberto Arroyo, Luis M. Bergasa, Rafael Barea, Elena López

**Affiliations:** 1 Department of Automation and Industrial Control, National Polytechnic, EC170135 Quito, Ecuador; E-Mail: andres_cela@yahoo.com; 2 Department of Electronics, University of Alcalá, Alcalá de Henares, E-28871 Madrid, Spain;E-Mails: roberto.arroyo@depeca.uah.es (R.A.); bergasa@depeca.uah.es (L.M.B.);barea@depeca.uah.es (R.B.); elena@depeca.uah.es (E.L.)

**Keywords:** low-cost, teleoperation system, humanoid robot, MechRc, Arduino Fio, ZigBee RF, Kalman filter, fuzzy balance control

## Abstract

Humanoid robotics is a field of a great research interest nowadays. This work implements a low-cost teleoperated system to control a humanoid robot, as a first step for further development and study of human motion and walking. A human suit is built, consisting of 8 sensors, 6 resistive linear potentiometers on the lower extremities and 2 digital accelerometers for the arms. The goal is to replicate the suit movements in a small humanoid robot. The data from the sensors is wirelessly transmitted via two ZigBee RF configurable modules installed on each device: the robot and the suit. Replicating the suit movements requires a robot stability control module to prevent falling down while executing different actions involving knees flexion. This is carried out via a feedback control system with an accelerometer placed on the robot's back. The measurement from this sensor is filtered using Kalman. In addition, a two input fuzzy algorithm controlling five servo motors regulates the robot balance. The humanoid robot is controlled by a medium capacity processor and a low computational cost is achieved for executing the different algorithms. Both hardware and software of the system are based on open platforms. The successful experiments carried out validate the implementation of the proposed teleoperated system.

## 1. Introduction

For decades, many research works have studied different methods to reproduce the movements of a person in electromechanical and robotic systems. These methodologies allow not only to replicate but also to improve and refine these movements for different uses for the welfare of humanity The teleoperation provides protection and increases the maneuverability of a huge variety of human-operated machines. Some examples are chemical, construction and mining industries and medicine. In the latter case, precise and reliable robotic systems must assist surgeons.

Currently, improved control systems for biped walking robots has attracted an increase research interest. One approach is to model and reproduce human-like walking given the angular velocity and acceleration measured by gyroscopes and accelerometers installed on the legs of a person [1].In the work by Nakazawa *et al.*, [2] an artificial vision system composed of 8 cameras captures dancing human movements to be imitated by a robot. In [3], a camera system is presented too, where Boesnach *et al.* introduce the concept of movement oriented to context. This is a third type of movement coming from the combination of task-oriented and position-oriented ones. Besides, they employ a trajectory generator and classifier to improve the guidance control of the robot depending on its position, the goal task and the environment. All these techniques replicate basic movements like lines, circles and their corresponding parameters. However, a high number of variables is required, increasing the complexity and computational requirements of these systems.

Due to this great interest in humanoid robotics and teleoperation, several robots are designed for R&D in specialized labs and universities. As an example, the Honda robot Asimo [4] for helping people in basic home duties and used as a research platform to improve motion algorithms and environment interaction too. Nao [5], a smaller humanoid robot, is widely employed internationally like in Robo-Cup competition [6]. However, their price is too high for reduced budgets and other cost-effective options exist, e.g., Robonova [7], Bioloid [8] or MechRc [9].

Recently, several low-cost humanoids platforms are available in the consumer market, where they are sold as high quality toys for personal entertainment. Nevertheless, they can also be employed as cost-effective alternatives for research projects which can lead to promising results. As an example, in [10], the Robosapien V2 toy is programmed to observe and imitate human gestures in order to learn them. The system comprises several sensory devices, such as a dataglove and a monocular camera. Similarly to their approach, we trust on the development of low-cost robotic systems to reach the public. Thus, overcoming the high cost of more advanced systems currently under research. Although, these enhanced systems will be feasible in the future.

This article presents the teleoperation of a MechRc robot [9] and the implementation of a nonlinear balance control system based on open-source and open-hardware platforms. A mechanical suit is built comprising several sensors that capture the person movements and efficiently transmit the measurements to the robot. The MechRc is an educational robot widely used to teach children and teenagers in UK schools. To our knowledge, this is the first time that MechRc is employed in a research project for the implementation of a teleoperated system. This humanoid is selected for this work due to its versatility, agility and low price.

The optimization of nonlinear systems is a computationally expensive task. Those systems presenting a high degree of freedom, such as a humanoid robot [1,11], are seriously affected. A fuzzy control approach [12,13] is nonlinear and yields a quick response that allows a real time implementation. In our case, every sensor in the teleoperator suit is treated as a joint variable for computation efficiency, in contrast to Cartesian variables defined in [11]. Finally, we also employ an additional accelerometer to control the stability of the robot by using a fuzzy control approach and a Kalman filter [14] to reduce the noise from this sensor.

The rest of the article is organized as follows: In Section 2 the suit, movement sensors, wireless transmission module and data frame are presented. Section 3 describes the implemented control system, which consists of servomotors, balance feedback, Kalman filtering and fuzzy control. Finally, several results are discussed in Sections 4 and 5 concludes the main aspects of our work.

## 2. Tele-Operator Suit

The teleoperation suit has 6 linear resistive sensors on the legs and 2 accelerometers on the arms. A microcontroller board Arduino Fio [15] provides the computational resources for the acquisition of sensors data, their digital conversion and the posterior transmission through a wireless module based on ZigBee technology [16]. The MechRc robot receives this data, which is forwarded to the servo motors in the robot.

### 2.1. Resistive Sensors and Accelerometers

**Figure 1.**
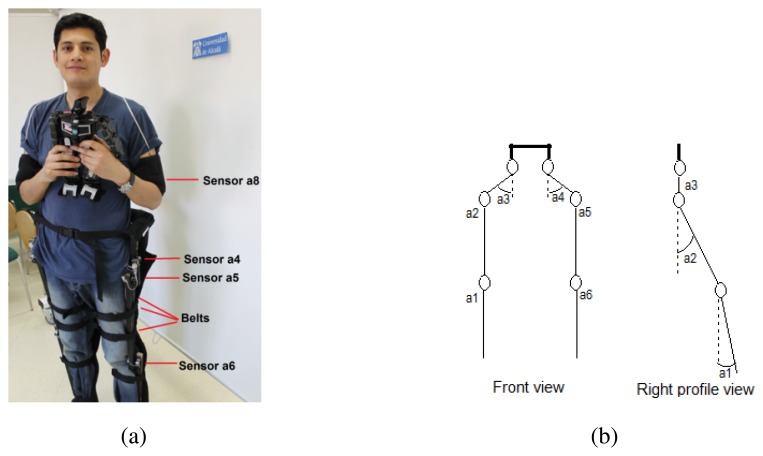
Teleoperator suit. (**a**) Suit on a person. (**b**) DoF of the joints.

Figure 1(a) shows the developed teleoperator suit. The Arduino Fio microcontroller installed on the suit provides 8 analog inputs that are used to capture data from the sensors. Due to this limitation in the number of channels of the ADC, only 8 sensors can be installed on the teleoperator suit and priority is given to the legs. Figure 1(b) depicts the degrees of freedom (DoF) at each joint of the legs. The thigh points have two DoFs while each knee joint has only one. The sensors in these joints measure different angles: *a*1 and *a*6 the knees flexion, *a*2 and *a*5 the legs rise up and *a*3 and *a*4 the legs opening.

Concerning the accelerometers in the bracelets, they have 3 degrees of freedom, although only the measurement of the shoulder angular movement with respect to frontal horizontal axis is considered, *i.e.*, movement parallel to sagittal plane, flexion/extension of arms with respect to shoulder.

Liu *et al.* [1] proposed a suit with a higher number of accelerometers and gyroscopes on the joints allowing more variables in the model to characterize yaw, roll and pitch variations. However, higher computational resources are required, which also means more power consumption. Our approach is a specific implementation for a fast 8 bit microprocessor that needs only a 3.3 volts battery.

### 2.2. Data Wireless Transmission

The digital outputs from the ADC are transmitted to a receiver integrated on the humanoid robot employing a wireless XBee module [17] attached to the Arduino Fio board. Considering that a low bandwith is needed for the radio communication channel between the teleoperator suit and the robot, ZigBee technology [16] has been chosen. Its low power requirements, low latency and low cost offers a reliable serial communication between the two devices at distances around 10 meters.

Given the linear response of the sensors and assuming Gaussian noise of very low amplitude, the relationship between sensors voltages and the angle of inclination of every joint can be easily and robustly computed. The output data from the ADC embedded on the Arduino Fio board has a resolution of 10 bits, which are reduced to 8 bits for saving reasons and mapped to the angle variation range of the servo motors: 0° – 179°. Thus, providing enough resolution to get steps of 1° in order to control them.

On the other hand, we propose a binary codification of the angle values, such that a byte encodes each angle (0° – 179°). Then, the resultant data frame for the 8 sensors has a size of 9 bytes (1 of them representing the frame header). Considering a serial communication channel of 57,600 bauds, the data frame transmission takes 1.56 ms.

## 3. Robot Control System

In this work, the humanoid robot MechRc [9] is employed to replicate the movements of the teleoperator suit described in the previous section. The hardware of the robot control system consists of a data reception module based on ZigBee technology, several servo motors integrated on the robot, a balance sensor (accelerometer) and a control board (Arduino Fio).

In the next subsections the servo motors and the balance feedback are presented. The Kalman filtering and the fuzzy algorithm proposed for robot stability control are described too.

### 3.1. Robot Servo Motors

**Figure 2.**
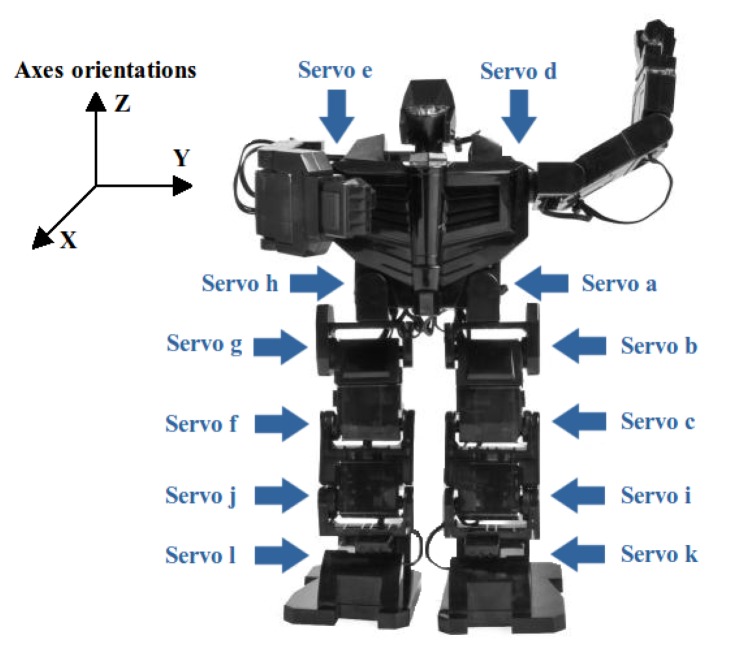
Location of robot servo motors and reference axes orientation.

**Figure 3.**
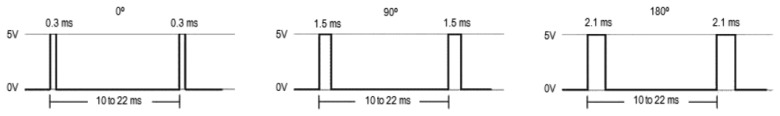
PWM control signals for a servo.

The humanoid robot movements are performed by a set of 12 actuators (see Figure 2) managed by an Arduino Fio microcontroller [15]. The servo control signal is a PWM (Pulse-Width Modulation), as shown in Figure 3, where there are three examples for 0°, 90° and 180°.

Each servo motor starts in a known position and is internally regulated by a PID (Proportional-Integral-Derivative) controller with resistive feedback. On the other hand, the states of the servos are unknown and cannot be used for the stability control. However, the whole robot state can be estimated measuring the initial positions and the angular increments.

### 3.2. Balance Feedback and Kalman Filtering

Replicating the person movements on the humanoid robot requires a balance control. The microcontroller actuates on the robot servo motors according to the measurements of the sensors in the suit. The direct execution of commands that include knee flexion, causes a loss of robot's stability and its consequent fall. Thus, a global balance control is needed.

**Figure 4.**
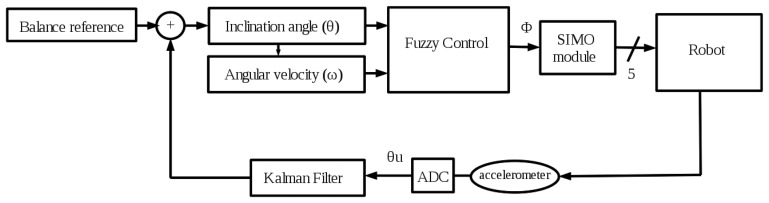
Balance control process in the frontal plane of the robot.

The balance feedback depicted in Figure 4 is done through the measurement provided by an accelerometer located in the robot's back. The state vector of the sensor contains 3-axes orientations (see Figure 2) called Euler angles [18]. However, in this work the needed axes are the X axis for the stability control in the frontal plane and the Y axis for a simple sagittal plane control of the robot balance. From these linear axes, their corresponding inclination angles are calculated using trigonometrical basic reasoning.

The balance control system is based on a Takagi-Sugeno fuzzy controller [19], which is sensitive to noisy inputs. Due to this, it is necessary to filter the sensor signal to improve the response of the controller. For this task, a Kalman filter [14] is employed, which is commonly used in related works to directly estimate the orientation [18] or correct small misalignments in accelerometers.

The X axis data includes a high Gaussian noise that must be lessened to obtain a reliable measurement of the inclination angle (*θ*) and the angular velocity (*ω*) in the frontal plane of the robot. This is achieved applying a Kalman filter, as it is shown in Figure 4. On the other hand, the measurement from the Y axis (*γ*) has a low noise and it does not require any filtering step. Then, a simpler control is used in the sagittal plane consisting on a fixed maximum inclination angle limiter. If *γ* is higher than this bound (empirically adjusted to 30°) some constant corrections are made on servo motors *a* and *h* (see Figure 2).

**Figure 5.**
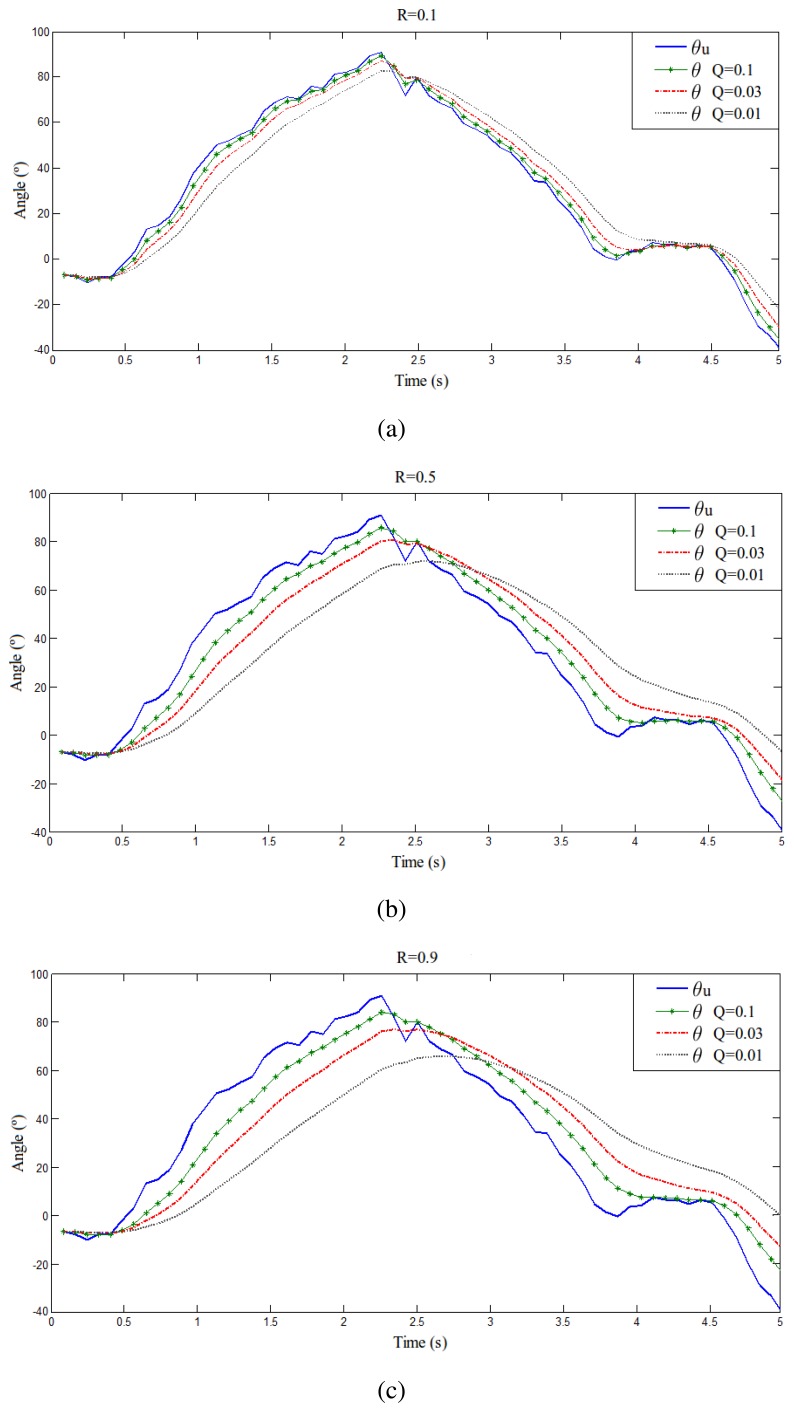
Comparative study of Kalman filter response for Q and R determination. (**a**) R = 0.1; (**b**) R = 0.5; (**c**) R = 0.9.

According to Kalman's formulation [14], the Q and R noise covariance matrices parameters are adjusted empirically [20]. It must be noted that the measurements from the accelerometer are slightly smoothed by an integrated hardware filter. Nevertheless, in order to achieve superior filter performance, Kalman method is applied. Figure 5 shows a series of plots of a sensor measurement from the accelerometer in the back of the robot and its filtered version for different *Q* and *R* values. As it can be seen, in Figure 5(a) there is an overfitting and in Figure 5(c) there is too much filtering and low tracking. Then, after these experiments, the selected values in Figure 5(b) for *R* and *Q* are 0.5 and 0.03, respectively. It is a trade-off between filter response delay, the level of signal smoothness and the tracking performance. Besides, these values account for the global system reponse. They allow to adjust the control delay with the mechanical delay, thus the execution of movements is smooth and correct.

Regarding to the deterministic matrices of the system, the following values are defined: *A* = 1; *B* = 0 and *C* = 1. *A* is the updating matrix depending on the previous state, *B* is the updating matrix depending on the input state and *C* relates the state with the measurement.

The Kalman filter initialization is set to *x*_0_ = *θu*_0_ and *P*_0_ = *Q*, where *x*_0_ is the initial state, *θu*_0_ is the initial unfiltered inclination angle and *P*_0_ is the initial error covariance matrix.

Considering the previous defined matrices and the initial conditions, the Kalman implementation featured for the robot can be summarized in the formulas below, where *x*′ is the state prediction, *x̂* is the state update and the filtered *θ* is considered as the Kalman output *x̂_k+_*_1_:

(1) $$\begin{array}{cc} x_{k+1}^{\prime }={\hat{x}}_{k} & \text{State prediction} \end{array}$$

(2) $$\begin{array}{cc} P_{k+1}^{\prime }=P_{k}+0.03 & \text{Error covarience prediction} \end{array}$$

(3) $$\begin{array}{cc} K_{k+1}=P_{k+1}^{\prime }*{\left[P_{k+1}^{\prime }+0.5\right]}^{-1} & \text{Gain estimation} \end{array}$$

(4) $$\begin{array}{cc} y_{k+1}=\theta u_{k+1} & \text{Sensor data reading} \end{array}$$

(5) $$\begin{array}{cc} {\hat{x}}_{k+1}=x_{k+1}^{\prime }+K_{k+1}[y_{k+1}-x_{k+1}^{\prime }] & \text{State update} \end{array}$$

(6) $$\begin{array}{cc} P_{k+1}=(1-K_{k+1}C)*P_{k+1}^{\prime } & \text{Error covarient update} \end{array}$$

### 3.3. Fuzzy Control of the Robot Balance

The robot balance control is performed by a fuzzy algorithm based on Takagi-Sugeno methodology [19]. This controller presents a low time response because of the definition of a set of simple functions that require low processing resources. Hence, it is a suitable method for real time purposes [21].

Fuzzy sets of input, output and the set of rules are defined based on prior knowledge of system behavior. More specifically, the two inputs for our fuzzy system correspond to the filtered frontal plane inclination angle (*x* = *θ*) and the angular velocity (*ω*) computed with Equation (7). The output of the fuzzy controller represents an angular compensation (*ϕ*) to keep robot balance.

(7) $$\omega =\frac{\Delta \theta }{\Delta t}=\frac{\theta_{k+1}-\theta_{k}}{\Delta t}$$

**Figure 6.**
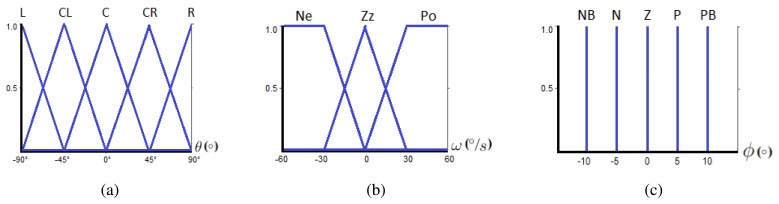
Fuzzy subsets for inputs and output. (**a**) *θ* subsets, (**b**) *ω* subsets, (**c**) *ϕ* subsets.

Figure 6(a,b) shows the fuzzy sets for each input. The measurements from the accelerometer are in the range from −90° to 90° (horizontal axis on Figure 6(a)). After several tests, it has been observed that sharp changes in tilt angle can vary from −50 to 50 °/*s*. Hence, a safety range from −60 to 60 °/*s* has been considered for input *ω* (horizontal axis on Figure 6(b)).

As it can be observed, the subsets for the linguistic variable *θ* are all triangular due to real time implementation. The subsets of *ω* have two trapezoidal memberships and a triangular central one, which is also a very common format [12]. The letters on top of the input subsets stand for *L* = *Left, CL* = *Center Left, C* = *Center, CR* = *Center Right, R* = *Right, Ne* = *Negative, ZZ* = *Zero and Po* = *Positive*.

This work proposes a zero order Takagi-Sugeno model, which is a kind of Mandami Fuzzy control where the consequent of the rule is a singleton function. Thus, the output function is a constant characterized by a set of Dirac deltas in the fuzzyfier. The range of the output fuzzy set is from −10° to 10° as depicted in Figure 6(c), where *NB* = *Negative B, N* = *Negative, Z* = *Zero, P* = *Positive, PB* = *Positive B*.

According to a singleton model, the rules are built upon Equation (8). Besides, it is a completeness system because the number of rules (*i*) is 15 which results from all the possible combinations of the input linguistic variables. In our approach, the rules have been established in an experimental way. However, some authors apply Genetic Algorithms or other heuristics for tuning the rules and membership functions [22].

(8) $$R^{i}:\text{IF}\;f(x_{1}\;\text{is}\;A_{1},\ldots ,x_{k}\;\text{is}\;A_{k})\text{THEN}\;y=\delta_{\text{m}}$$

The conjunctive operator **AND** that connects fuzzy input variables has been set to **MIN** (*r_x_* = **min**(*θ*,*ω*)) because it is usually employed for practical cases, while the **PROD** is used in stability studies [23]. Additionally, for the aggregation of N equal consequents a **MAX** $\left(u_{R,\delta_{k}}=\max[V_{i-1}^{N}r_{i}\to Y_{\delta k}]\right)$ is selected. Assuming the following definitions *x*_1_ ≔ *θ*, *x*_2_ ≔ *ω* and *y* ≔ *ϕ*, the 15 rules are enumerated below:

$$\begin{array}{cccccccccc} \text{R}1 & \text{If} & \theta \;\text{is} & \text{L} & \text{and} & \omega \;\text{is} & \text{Ne} & \text{Then} & \varphi \;\text{is} & \text{PB}\\ \text{R}2 & \text{If} & \theta \;\text{is} & \text{L} & \text{and} & \omega \;\text{is} & \text{ZZ} & \text{Then} & \varphi \;\text{is} & P\\ \text{R}3 & \text{If} & \theta \;\text{is} & \text{L} & \text{and} & \omega \;\text{is} & \text{Po} & \text{Then} & \varphi \;\text{is} & P\\ \text{R}4 & \text{If} & \theta \;\text{is} & \text{CL} & \text{and} & \omega \;\text{is} & \text{Ne} & \text{Then} & \varphi \;\text{is} & P\\ \text{R}5 & \text{If} & \theta \;\text{is} & \text{CL} & \text{and} & \omega \;\text{is} & \text{ZZ} & \text{Then} & \varphi \;\text{is} & P\\ \text{R}6 & \text{If} & \theta \;\text{is} & \text{CL} & \text{and} & \omega \;\text{is} & \text{Po} & \text{Then} & \varphi \;\text{is} & Z\\ \text{R}7 & \text{If} & \theta \;\text{is} & \text{C} & \text{and} & \omega \;\text{is} & \text{Ne} & \text{Then} & \varphi \;\text{is} & P\\ \text{R}8 & \text{If} & \theta \;\text{is} & \text{C} & \text{and} & \omega \;\text{is} & \text{ZZ} & \text{Then} & \varphi \;\text{is} & Z\\ \text{R}9 & \text{If} & \theta \;\text{is} & \text{C} & \text{and} & \omega \;\text{is} & \text{Po} & \text{Then} & \varphi \;\text{is} & N\\ \text{R}10 & \text{If} & \theta \;\text{is} & \text{CR} & \text{and} & \omega \;\text{is} & \text{Ne} & \text{Then} & \varphi \;\text{is} & Z\\ \text{R}11 & \text{If} & \theta \;\text{is} & \text{CR} & \text{and} & \omega \;\text{is} & \text{ZZ} & \text{Then} & \varphi \;\text{is} & N\\ \text{R}12 & \text{If} & \theta \;\text{is} & \text{CR} & \text{and} & \omega \;\text{is} & \text{Po} & \text{Then} & \varphi \;\text{is} & N\\ \text{R}13 & \text{If} & \theta \;\text{is} & \text{R} & \text{and} & \omega \;\text{is} & \text{Ne} & \text{Then} & \varphi \;\text{is} & N\\ \text{R}14 & \text{If} & \theta \;\text{is} & \text{R} & \text{and} & \omega \;\text{is} & \text{ZZ} & \text{Then} & \varphi \;\text{is} & N\\ \text{R}15 & \text{If} & \theta \;\text{is} & \text{R} & \text{and} & \omega \;\text{is} & \text{Po} & \text{Then} & \varphi \;\text{is} & \mathrm{NB} \end{array}$$

Let us define the following set of variables on Equations (9–13) represented by the notation *u_R,δk_* and corresponding to the singleton consequents *PB*, *P*, *Z*, *N* and *NB*.

(9) $$\text{pbr}=r_{1}\to Y_{\delta_{1}}$$

(10) $$\text{pr}=\text{max}(r_{2},r_{3},r_{4},r_{5},r_{7}\to Y_{\delta_{2}})$$

(11) $$\text{zr}=\text{max}(r_{6},r_{8},r_{10}\to Y_{\delta_{3}})$$

(12) $$\text{nr}=\text{max}(r_{9},r_{11},r_{12},r_{13},r_{14}\to Y_{\delta_{4}})$$

(13) $$\text{nbr}=r_{15}\to Y_{\delta_{5}}$$

Then, the result from fuzzy control in order to compensate the servo motors is given by Equation (14).

(14) $$\phi =\frac{\left(\text{pbr}.\text{PB})+(\text{pr}.P)+(\text{zr}.Z)+(\text{nr}.N)+(\text{nbr}.\text{NB}\right)}{\text{pbr}+\text{pr}+\text{zr}+\text{nr}+\text{nbr}}$$

Finally, this angular correction is applied to different servo motors depending on the right or left knee flexion in order to achieve the goal of keeping robot's stability This function is carried out by the SIMO (Single Input Multiple Output) module in Figure 4. Besides, Table 1 collects the joints compensations to be applied on the servo motors (check Figure 2 to locate each servo on the robot). The servo motors *i* and *j* correspond to the ankles of the robot and they are set to the same angle as the corresponding knee in order to have coherent angles in the segment that links them. The constant values (c*) on the table have been empirically determined.

### 3.4. Fuzzy Control Simulation

**Figure 7.**
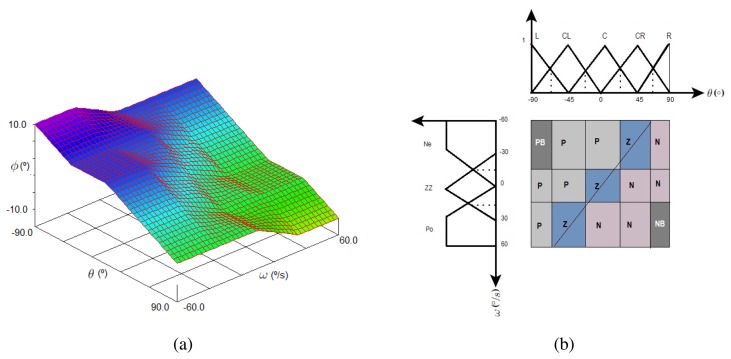
3D output surface and states space. (**a**) 3D output surface obtained with XFuzzy tools. (**b**) States space and transition line.

This system has been previously analyzed using XFuzzy tools resulting in the control surface of Figure 7(a). The surface shows the relation between fuzzy input variables *θ* and *ω* and the output *ϕ*, which can vary from 10° to −10°, being 10° when the *θ* fuzzy input is −90°. For this case, the other input variable (*ω*) can be at its maximum or minimum values. This indicates that the control system is correcting the error as much as possible. Furthermore, it can be observed the desired system behavior: when the angle and angular velocity variables are negative, the fuzzy algorithm returns a positive output to compensate and keep the robot balance and vice versa.

Figure 7(b) depicts the transition line in the states space allowing an early error detection at the moment of rules design. Additionally, it can be observed the distribution of consequents of the rules along the states space.

**Figure 8.**
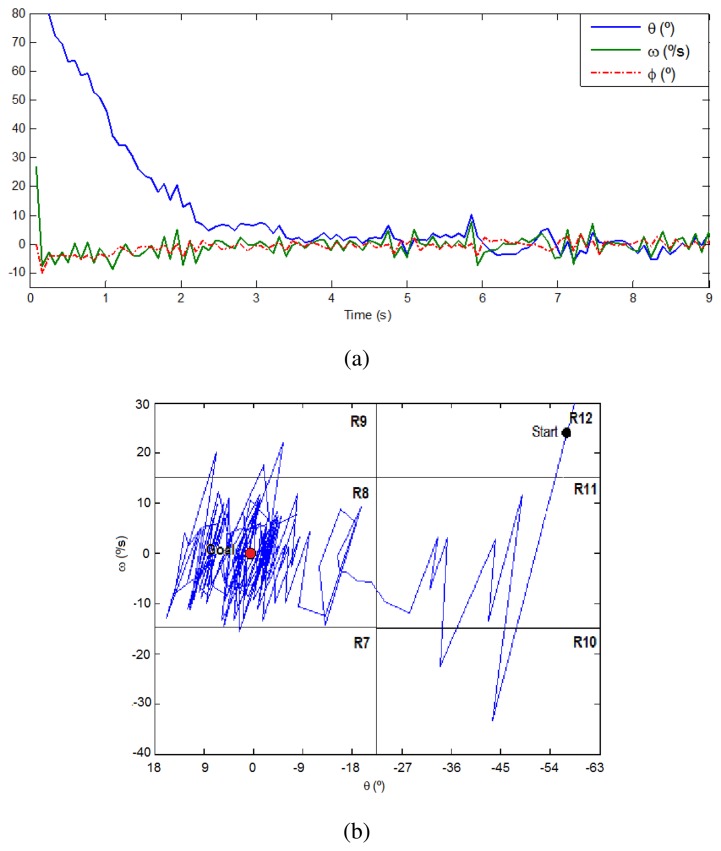
Results of a simulation exercise. (**a**) Input signals *θ* and *ω* and the resulting angularcorrection *ϕ*. (**b**) Linguistic trajectories.

Moreover, Figure 8(a) presents a plot of the inputs and output signals in a simulation exercise of angular correction. The robot is stabilized around the angle of 0° and an angular velocity of 0 °/*s*, through the application of a mild angular correction (−10°, 10°). The trajectory along the linguistic variables during this simulation is depicted on Figure 8(b). The starting point is randomly set on R12 (58′5°, 25 °/*s*) and the goal is on R8 (0°, 0 °/*s*). There is a clear oscillation until reaching the stability vector in the center of R8. Several additional simulations have been done configuring different points of the states space and the results were similar. Then, these experiments validate the successful correction performed by the proposed fuzzy control algorithm.

## 4. Results and Discussion

The 8-bit Arduino Fio microcontroller [15] is based on the ATmega328P processor at 8 MHz and a flash memory of 32 KB. Considering this hardware, Table 2 summarizes the different algorithm delays of our approach and the total processing time on the robot. Furthermore, considering the teleoperator suit and data transmission, the total teleoperation time is 517 ms, which counts the delay of a knee flexion exercise since the movement starts in the teleoperator suit until the robot starts the replication. This time is better than the teleoperation development in [5], which takes approximately 1.5s with a more expensive system.

**Figure 9.**
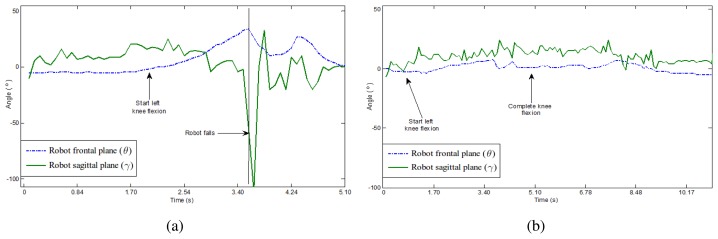
Angular variations in frontal and sagittal planes for a knee flexion exercise. (**a**) No balance control. (**b**) Balance control active.

Figure 9 represents in two plots the frontal and sagittal angular variations in a knee flexion exercise with and without fuzzy control. It can be observed on the left plot, without balance control, that the robot falls down. As far as the angular variation in both planes increases, the robot starts performing the knee flexion but it finally fails, which is reflected by a sharp change on both curves. Right plot corresponds to the same exercise but the balance control is active and prevents the robot to fall.

**Figure 10.**
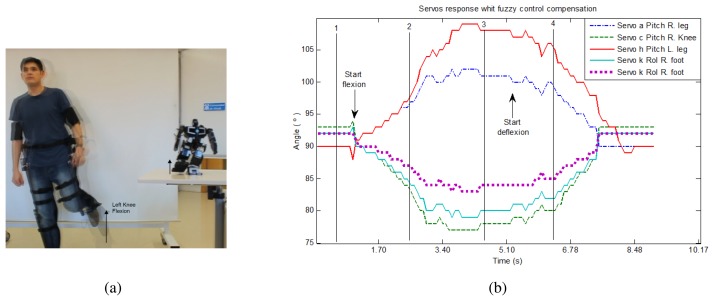
Left knee flexion exercise. (**a**) Human pose in left knee flexion and robot mirrored replication. (**b**) Compensated angles of the involved servomotors in the left knee flexion.

The following sequence of knee flexion is a test experiment that proves the fuzzy control of the robot balance. This sequence consist of idle, starting knee flexion, complete flexion and idle stages. A sample image is shown in Figure 10(a). Besides, Figure 10(b) includes a plot of the involved servo motors response with fuzzy control compensation.

**Figure 11.**
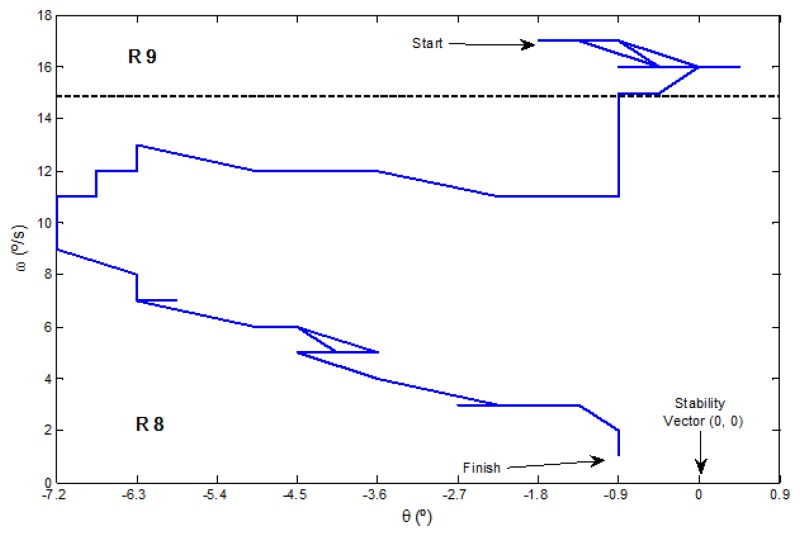
Results of linguistic trajectories in a real left knee flexion exercise.

During the knee flexion exercise, the trajectory along the linguistic variables starts in R9 space and crosses to R8, until it finishes in the stability vector placed around the angle of 0° and an angular velocity of 0 °/*s*, as can be seen in the real example presented in Figure 11.

**Figure 12.**
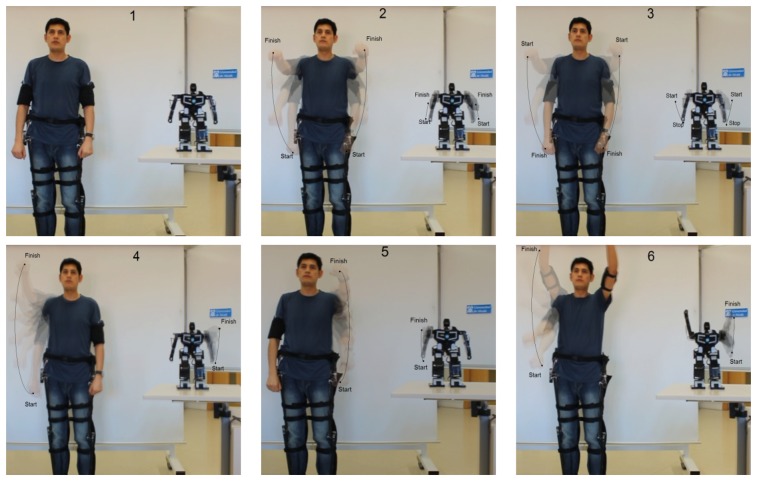
Sample movement sequence. (**a**) Down; (**b**) Moving up; (**c**) Moving down; (**d**)Right shoulder up; (**e**) Left shoulder up; (**f**) Both shoulders up.

**Figure 13.**
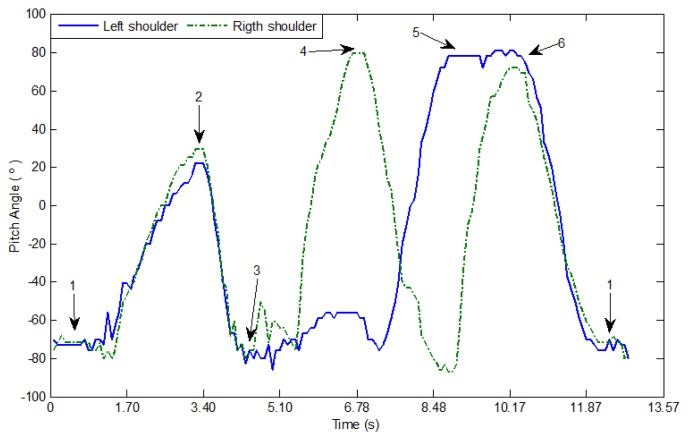
Angular measurement of the rotation of sagittal plane on the left and right shoulders when the teleoperator is moving the arms up and down.

In other tests, the teleoperator follows a sequence of arms movements up and down (Figure 12). The accelerometers in the arms measure the angular change of the sagittal plane with respect to the frontal horizontal axis on each shoulder. A plot of both variations is displayed in Figure 13.

## 5. Conclusions

In this work, the teleoperation of a MechRc robot and the implementation of a nonlinear balance control based on open-source and open-hardware platforms have been presented, obtaining a complete low-cost solution for this kind of systems. A human suit with 8 linear sensors including a processing unit based on Arduino Fio microcontroller has been designed. The humanoid robot receives sensors measurements from teleoperator movements employing ZigBee technology.

We have demonstrated the reliability of the system by the implementation of a robust and efficient control system, which includes a robot stability control placing an accelerometer on its back. A Kalman filter reduces the Gaussian noise produced by this sensor and it is easily calibrated depending on the standard deviation of the system. This makes feasible the use of a fuzzy controller for the robot balance that allows the robot to successfully replicate the suit movements without falling down. The inputs of the fuzzy algorithm, *i.e.*, the robot inclination angle and angular velocity, take into account not only if the robot tends to fall but also its speed, making the system more robust and yielding promising results as a first approach.

In addition, a low computational cost with a medium capacity processor is achieved. A single processor is responsible for controlling the robot servo motors, which implies the following main tasks: measurement acquisition and reception, Kalman filtering, fuzzy algorithm execution and balance adjustment. The operation delay is around 500 ms, which can be improved using faster math and faster processors (like ARMs).

Incrementing the number of sensors on the teleoperator suit and replacing the linear ones by inertial sensors would allow higher flexibility for capturing teleoperator movements. For the future, we also intend to implement the control in two axes, applying the inverted pendulum method and obtaining differential equations that lead to more natural walking of the humanoid robot. Furthermore, we plan to obtain rules and membership functions of the fuzzy controller in an automatic way. There is also of interest to improve the stability control of the robot including a formal stability analysis.

## Figures and Tables

**Table 1.** Joints compensations for each servo motor depending on knee flexion.

**Left Knee Flexion**			**Right Knee Flexion**		

**Joint**	**Compensator**	**Scale**	**Joint**	**Compensator**	**Scale**
a servo	+	c1	a servo	+	c1
h servo	+	c1	h servo	+	c1
k servo	-	c2	k servo	-	c1
1 servo	-	c3	1 servo	-	c3
f servo	-	c1	c servo	-	c2

**Table 2.** Processing time of the principal functions.

**Algorithm**	**Time**
Servos control	1.560 ms
Kalman filtering	0.660 ms
Fuzzy control	0.276 ms
Remaining subfunctions	2.304 ms
Total algorithms	4.800 ms
ADC channels delay	80.000 ms
Total time	84.800 ms
